# 

**Published:** 1916-12

**Authors:** 


### CONTENTS.

ORIGINAL COMMUNICATIONS……375

EDITORIAL……393

SELECTIONS AND ABSTRACTS……394

BOOK REVIEW……408

